# Supplementary material for: Comparative multiomics analysis of cell physiological state after culture in a basket bioreactor
Source: Sci Rep. 2022 Nov 23;12:20161. doi: 10.1038/s41598-022-24687-4 (PMC9686226; doi:10.1038/s41598-022-24687-4)

3666077.1:16019511:16019586:+@NW_023666077.1:16019511:16019661:+@NW_023666077.1:16019887:1602[illegible]

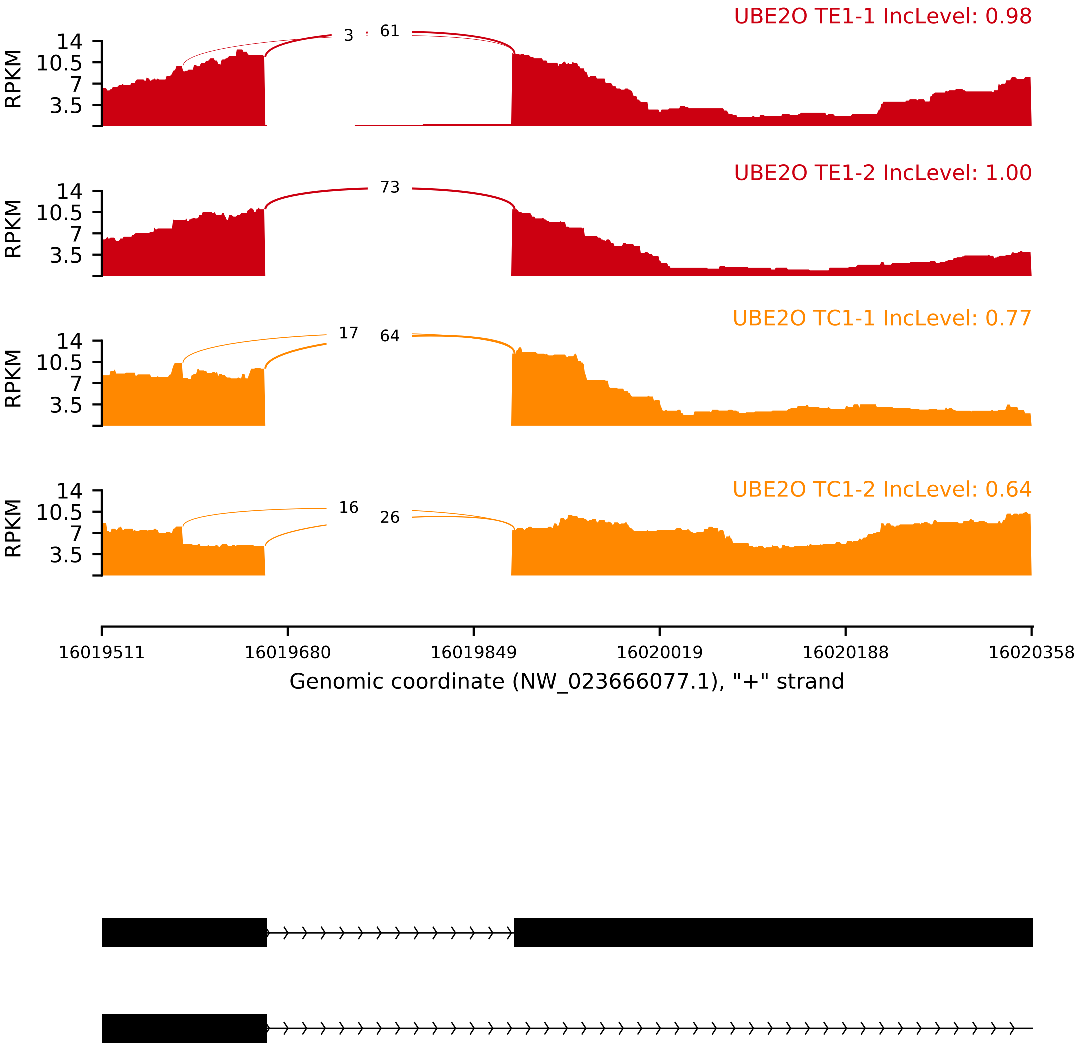

Supplement: Supplementary file 1 — Supplementary Information 1. [file 41598_2022_24687_MOESM1_ESM.zip › raw data/Transcriptomic raw data/7.AS/2.dasplot/TE1vsTC1/A5SS/17_UBE2O_NW_023666077.1_16019511_16019586_+@NW_023666077.1_16019511_16019661_+@NW_023666077.1_16019887_16020359_+.pdf]
